# Moderation of Housing-Related Factors on Psychological Capital–Job Embeddedness Association

**DOI:** 10.3389/fpsyg.2019.01618

**Published:** 2019-10-10

**Authors:** Tianzhou Ren, Xizan Jin, Muhammad Rafiq, Tachia Chin

**Affiliations:** School of Management, Zhejiang University of Technology, Hangzhou, China

**Keywords:** psychological capital, job embeddedness, housing-related factors, entrepreneurship, China

## Abstract

This study identified three critical housing-related factors (HRFs) as moderators of the relationships between employees’ psychological capital (PsyCap) and job embeddedness (JE) in China’s entrepreneurial environment. The hypotheses were tested with multiple hierarchical regression modeling, using the data collected from 312 employees in manufacturing organizations. The results demonstrated that HRFs (i.e., home ownership, housing price satisfaction, and contributions to housing funds) moderate the relationships between employees’ PsyCap and JE. Specifically, these relationships were stronger when the HRFs were high. Such results contribute to the PsyCap and JE literature by incorporating housing as an extrinsic life-aspect factor that might affect employees’ psychological state and thus their retention in works. Implications for the organization policies and directions for future studies were discussed.

## Introduction

In today’s rapidly changing and hectic entrepreneurial environment, positive psychological factors are becoming much more important because such resources help people cope with work stress and anxiety and thus make employees more proactive, innovative, and resilient (Chin et al., 2019; Rafiq and Chin, 2019).

Psychological capital (PsyCap) is one of the most significant positive psychological resources of an individual because it enables individuals to keep positive, hopeful, confident, resilient, and optimistic at the workplace (Luthans et al., 2007; Chen and Pan, 2019; Wang et al., 2019). Hu et al. (2018) indicated that PsyCap has been considered as an important antecedent of individuals’ wellness, health, and other behavioral outcomes in a performance-oriented work setting. Although a large body of research has demonstrated the effect of PsyCap on job-related behaviors (Luthans et al., 2007) and the significance of positive psychology in an entrepreneurial context is widely recognized, there have been limited studies addressing how individuals’ positive psychological strengths affect or reinforce their positive mental functioning. To fill this gap, this paper aims to investigate the impact of a critical mental force (i.e., PsyCap) on a crucial manifestation of positive psychological functioning (i.e., job embeddedness) in China.

Mitchell et al. (2001) introduced the concept of “job embeddedness” as the forces that retain people’s interest and energies in doing their jobs. These forces consisted of three dimensions: fit, link, and sacrifice. *Fit* is the extent to which an individual’s ability is compatible with the surrounding environment and organization. *Links* include the number of connections to individuals and groups within their organizations. *Sacrifice* is defined as what an individual would have to give up (i.e., perceived costs of social, psychological, and financial benefits) if they decide to leave their organization.

Despite a variety of antecedents of job embeddedness (JE) have been proposed, the association of PsyCap with JE has not been fully understood (Sun et al., 2012). In consideration of such a gap in existing literature, we aim to investigate the impact of PsyCap on JE.

Moreover, potential moderators, especially those from the off-the-job, external aspects, should be considered and incorporated as we study the PsyCap-JE relationships. The contingency theory (Donaldson, 2001) suggested that context does matter when looking into employees’ motivational and behavioral functions. Such an argument is explicitly supported in the PsyCap literature, too (Newman et al., 2014).

In such vein, we suggest incorporating critical environmental factors; especially those come in line with the logic of PsyCap and JE, into our examinations. Scholars have begun to emphasize the importance to investigate how key environmental factors shape employees’ psychological outcomes (Rafiq and Chin, 2019; Sung and Choi, 2019). Evidence reveals that home ownership and fund-related opportunities can raise residents’ satisfaction on social security whereby their social responsibility and sense of national identity can be largely enhanced (Saunders, 1990; Yeung and Howes, 2006; Groves, 2007). These studies also find a positive association among all these HRFs. But the existing research on HRF for China is limited. According to Chinese history, housing-related issues have been of particular importance to individuals’ positive psychological feelings in this context. We thus attempt to focus on investigating the moderation effects of HRF here.

China’s preference for HRF has a deep historical and socio-cultural background. Not China’s but also for everywhere, housing is the prime consumption item in their lifetime, and home is the setting where one finds satisfaction, refuge, and rest. But in China, the traditional culture of “having a house has a home” or “having a family and career is the aim of life” subtly makes many Chinese residents prefer to obtain housing ownership, which directly affects people’s sense of belonging and wellbeing-related outcomes (Yeung and Howes, 2006).

While the HRF can be regarded as a very important part of the social welfare system in contemporary China, it is expected that HRFs are widely recognized as critical environmental factors affecting Chinese people’s mental state and sense of wellbeing. We thus expect that the key HRF such as local house ownership, satisfaction toward housing prices, and the contribution to the national housing funds may moderate the psychological state-behavior mechanisms. To concern and make a contribution to the contextual thinking, we make the following hypotheses:

*Hypothesis 1*: Local house ownership positively moderates the relationship between psychological capital and job embeddedness in such a way that this relationship is stronger when the level of local house ownership is high than when it is low.

*Hypothesis 2*: Satisfaction toward local housing price positively moderates the relationship between psychological capital and job embeddedness in such a way that this relationship is stronger when the level of satisfaction toward local housing price is high than when it is low.

*Hypothesis 3*: The contribution to housing funds positively moderates the relationship between psychological capital and job embeddedness in such a way that this relationship is stronger when the level of contribution to housing funds is high than when it is low.

## Materials and Methods

### Samples and Procedures

The whole survey that lasted for about 6 months was conducted in four manufacturing organizations in Guangxi province of China. Before the formal date of collection, we carried out a pilot testing with a sample of 60 workers from a well-known manufacturing organization in Guanxi, so as to examine whether the overall survey was clear, thorough, and robust. After the pilot test, the investigators modified some items working according to the feedback from the participants. The formal survey was conducted in three large manufacturing firms in the same province. The researchers first contacted the human resource managers of all three organizations for their assistance to arrange an appropriate time and suitable venues to distribute the questionnaires. To control for extraneous influence, only the full-time employees were requested to participate in the survey, where confidentiality and anonymity were ensured. The human resource managers of all three firms helped us retrieve completed questionnaires at two separate time points. At time 1, participants completed the questionnaires on PsyCap, HRF, and demographics. After 3 months (time 2), the same participants were requested to complete the survey on the outcome variable, JE.

At time 1, a total of 380 surveys were distributed to the participants and 355 were returned yielding a response rate of 93.4%. Among them, 312 participants completed the time 2 questionnaires yielding a response rate of 87.8%. Finally, a total of 312 employees participated in this study, 260 were male (83.3%). The majority of the sample was male (83.3%) and had a undergraduate degree (41.7%). The age of respondents ranges from 19 to 45 years, with a mean age of 33.67 (SD = 0.70). Their average organizational tenure is 6.59 years (SD = 1.45).

### Measures

#### Psychological Capital

Psychological capital (PsyCap) short scale was used to measure PsyCap Luthans et al. (2007). It is a 12-item scale that originally reduced from the 24-item scale consisting of four dimensions of PsyCap: hope (4-item), efficacy (3-item), resilience (3-item), and optimism (2-item). Sample items include: “There are lots of ways around any problem” (hope), “I feel confident presenting information to a group of colleagues” (efficacy), “I usually take stressful things at work in stride” (resilience), “I am optimistic about what will happen to me in the future as it pertains to work” (optimism). Responses were on a 5-point Likert scale (1 = strongly disagree to 5 = strongly agree). In our sample, *α*s were 0.834 (hope), 0.825 (efficacy), 0.902 (resilience), and 0.854 (optimism). Overall, Cronbach’s *α* of employees’ PsyCap was 0.92.

#### Job Embeddedness

Job embeddedness was measured with a 7-item scale developed by Crossley et al. (2007). Sample items include: “I simply could not leave the organization that I work for” and “It would be difficult for me to leave this organization.” The participants were requested to respond on a 5-point Likert scale range from 1 (strongly disagree) to 5 (strongly agree). In the current research, the Cronbach’s *α* obtained was 0.94.

#### Housing-Related Factors

Housing-related factors were treated as dummy variables: (1) whether or not the respondents had owned a house (0 = no, 1 = yes), (2) whether or not the respondents had satisfied with the current house price (0 = no, 1 = yes), and (3) whether or not the respondents had contributed to the housing funds (0 = no, 1 = yes).

#### Demographic Variables

Gender, marital status, age, and education were applied as control variables in the data analysis.

### Analysis

The data were analyzed using statistical software’s SPSS 25.0. The Pearson bivariate correlation was used to explore the potential associations among variables. To examine the hypothesized moderation model, we used the SPSS macro PROCESS introduced by Hayes (2012). Additionally, we employed conventional methods for plotting simple slopes to understand moderation effects, at one standard deviation below and above the mean (Aiken and West, 1991).

## Results

### Correlational Analysis

Before testing the hypotheses, we examined the means, standard deviation, Cronbach’s *α* coefficients, and Pearson bivariate correlation among the study variables, as seen in Table 1. The Cronbach’s *α* coefficients were all greater than 0.70 (Nunnally and Bernstein, 1994). Results in Table 1 show that employees’ PsyCap was positively associated with JE (*r* = 0.26, *p* < 0.01). House ownership, house price-related satisfaction, and the contribution to housing funds were also positively related to employees’ JE (*r* = 0.18, *p* < 0.01; *r* = 0.11, *p* < 0.05; *r* = 0.20, *p* < 0.01), respectively. The results reflect a preliminary analysis of the anticipated associations.

**Table 1:** Means, standard deviations, Cronbach’s α, and Pearson bivariate correlations of the study constructs.

		1	2	3	4	5	6	7	8	9
1.	Psychological capital	(0.92)								
2.	Job embeddedness	0.26^**^	(0.94)							
3.	House ownership	0.54^**^	0.18^**^	–						
4.	STHP	0.45^**^	0.11^*^	0.57^**^	–					
5.	Contribution to house funds	0.38^**^	0.20^**^	0.56^**^	0.26^**^	–				
6.	Sex	0.07	0.21^**^	−0.15^**^	−0.04	0.11^*^	–			
7.	Marital status	−0.33^**^	−0.09	−0.16^**^	−0.09	−0.25^**^	0.44^**^	–		
8.	Age	−0.30^**^	−0.21^**^	−0.13^*^	−0.31^**^	−0.10	0.18^**^	0.76^**^	–	
9.	Education	−0.09	−0.11^*^	0.04	−0.29^**^	−0.16^**^	0.15^**^	0.14^**^	0.29^**^	–
	Mean	3.6	3.9	0.58	0.70	0.62	0.83	0.50	1.79	2.04
	Standard deviation	0.86	0.75	0.49	0.45	0.48	0.37	0.50	0.70	0.84

*N = 312; numbers on the diagonal represent Cronbach’s α of coefficients; STHP = satisfaction toward house price*.

* *p < 0.01*,

** *p < 0.05*.

### Hypotheses Testing

In this study, all the hypotheses were tested by using moderation examination. Specifically, we employed SPSS macro PROCESS to estimate moderations and obtain the bias-corrected bootstrapped confidence intervals (Hayes, 2012). The SPSS macro PROCESS runs each predictor construct individually, with added predictor constructs run as covariates. The model consists of employees’ PsyCap as the predictor construct (X), HRF as the moderator (W), and JE as the dependent (Y). For the analyses, all the continuous constructs were mean centered, a 95% bias-corrected percentile bootstrapped confidence interval (CI) method was used, and 5,000 bootstrap re-samples were produced for moderate examination.

**Figure 1:**
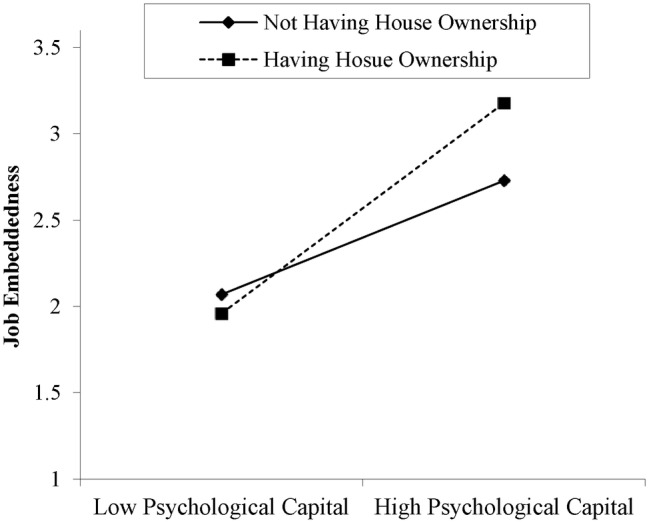
Moderation role of house ownership between psychological capital and job embeddedness.

In terms of *Hypothesis 1*, we proposed the moderation role of local house ownership on the association between employees’ PsyCap and JE. The results show that the local house ownership strengthened the association between employees’ PsyCap and JE (*β* = 0.144, *p* < 0.05). Furthermore, a follow-up simple slope analysis demonstrated that the positive association between employees’ PsyCap and JE was more pronounced among the employees who have local house ownership as compared to those who do not have local house ownership (see Figure 1). Hence, *Hypothesis 1* was fully supported.

**Figure 2:**
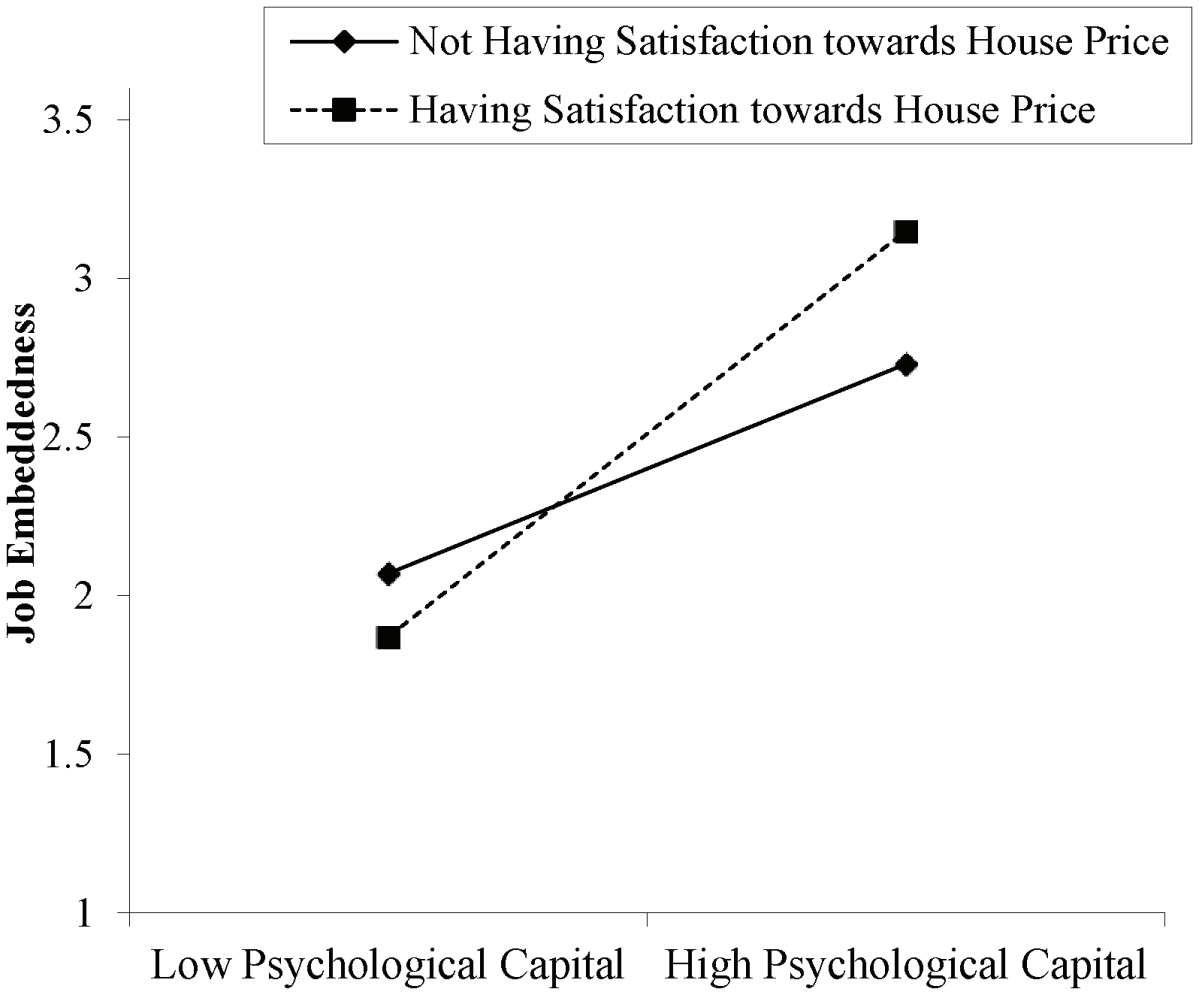
Moderation role of satisfaction toward house price between psychological capital and job embeddedness.

In supporting *Hypothesis 2*, the results show that the positive association between employees’ psychological and JE was positively moderated by satisfaction toward local house price (*β* = 0.261, *p* < 0.00). Additionally, as presented in Figure 2, employees’ PsyCap and JE were strengthened for those who feel satisfied toward local house price (see Figure 2) as compared to those who do not feel satisfied toward local house price. So, *Hypothesis 2* was supported.

**Figure 3:**
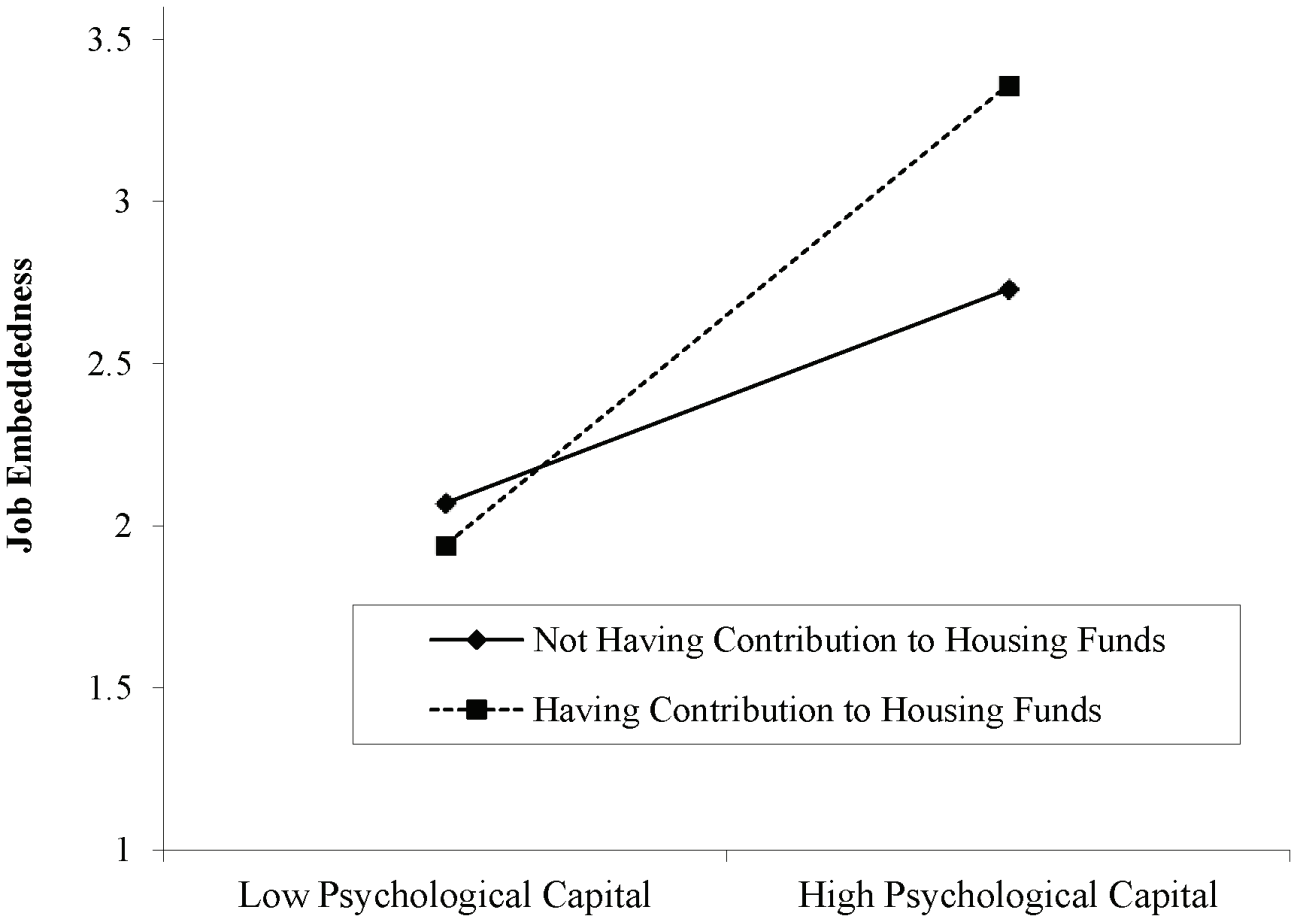
Moderation role of housing contribution to housing funds between psychological capital and job embeddedness.

Finally, in supporting of *Hypothesis 3*, the results show that the association between employees’ PsyCap and JE was also positively moderated by the contribution to housing funds (*β* = 0.304, *p* < 0.00). Additionally, as presented in Figure 3, employees’ PsyCap and JE were strengthened for those who have a local housing funding-related benefit as compared to those who do not have local housing funding-related benefit (see Figure 3).

## Discussion

All three hypotheses are fully supported; this current study thus examines the moderating role of HRF on the relationships between PsyCap and JE among manufacturing employees in an emerging entrepreneurial environment, China. By integrating contingency theory, our findings contribute to employees’ PsyCap research by suggesting that the employees’ PsyCap-JE linkage is affected by different housing-related factors, thus providing context-specific boundary conditions for this relationship. Overall, we make several theoretical and practical contributions to the existing body of knowledge in the following paragraphs:

First, we unveil the critical role of local house ownership in intervening the underlying linear mechanisms between employees’ PsyCap and JE, such that the relationship is strengthened for those who have their own house. Prior studies have consistently found that an accessible and supportive residential environment for individuals has a positive influence on their overall employees’ wellbeing (Rioux, 2005; Oswald et al., 2007; Ahn and Lee, 2016). This is an important extension to the employees’ PsyCap and JE literature because previous research has not taken into account the possibility that house ownership-related factors could interpret embeddedness differently.

Second, our findings show that the housing-related factor and employees’ satisfaction toward local housing price moderate the relationship between their PsyCap and JE, such that the relationship is stronger for those who feel satisfied toward local housing price. Our results to a certain extent echo prior research. Berry and Dalton (2004) reported that continuously increasing house prices have become the main reason for increasing the average wage level and social insurance of local people in major cities of Australia. Chen and Ellinger (2011) manifested that in no region of the world is the business environment more competitive and dynamic than in the hastily transforming and developing a nation of Republic of China where even during the global recession, the economy was growing exponentially and the housing price was continuously rising. Ren and Hu (2016) also argued that the living and housing conditions in China have changed substantially after the Housing Reforms and housing price have become a major concern for all Chinese families. Viewed from this angle, we further argue that all firms in China should pay more attention to local housing price as it may largely influence employees’ happiness at work and their willingness to stay or leave the organizations.

Third, we also found that the contribution to housing funds moderates the relationship between employees’ PsyCap and JE, such that relationship is stronger for those who have housing-related social insurance. Moreover, our findings show that a place that provides circumstances making individuals feel more psychologically attached is more attractive and individual tends to reside there longer and also feels more embedded. Viewed from this angle, we further suggest that Chinese firms may consider the contribution to housing funds as a motivation strategy to retain good local employees.

Final, the findings of this study also support a “contingency perspective” (Donaldson, 2001) to HR practices. These practices are “contingent practices” in which they are more strongly related to employee attitudes and behavioral outcomes. Support for such a “contingency perspective” is also provided by Chen and Ellinger (2011), who reported that a necessary condition for employees’ motivational and behavioral relationships is, depends on different critical environmental factors.

Findings provided are instructive to practitioners. Our findings reveal that individuals who have housing-related opportunities such as housing ownership, the higher level of satisfaction toward local housing prices, and contributions to housing funds as critical contingency factors that substantially change the relationship between employees’ PsyCap and JE at the workplace. As such, critical contingency factors can also assist organizations in understanding employee needs and accordingly taking appropriate measures to increase employees’ performance and retention. In particular, given the great importance of employees’ PsyCap and JE on firm performance (Chin, 2015; Rafiq and Chin, 2019), firms in China should put housing as a critical incentive to motivate Chinese employees, while counselors and educators could use these three HRFs to diagnose the problems individuals have during their career development process. For example, in China’s unique cultural context, these HRFs may be especially influential in increasing employees’ PsyCap as well as embeddedness and “make them feel wanted.”

Our study is not without limitations. First, given the research was conducted in the manufacturing industry in China, the generalizability of its findings to other societies or industries must be made with caution. As mention above, there is no country in the world that continuously makes progress in hastily changing, transforming, and developing a nation of Republic of China where the economy also keeps growing in the periods of recession (Chen and Ellinger, 2011). Therefore, our results might be unique to China and might not be applicable to other cultural contexts. Future research can be extended to include samples from other cultures or other industries for the aim of enhancing the external validity of the findings. Second, causal inference cannot be made due to the non-experimental and cross-sectional nature of the current research design. Although the moderation model established by us was guided by empirical and theoretical research, it is possible that there are inverse relations between the variables. Furthermore, though we controlled for several potential demographic variables, we are unable to ignore the effects of all demographic variables. Thus, we call for future research to use a longitudinal design to further validate our hypothesized relationships found in the present research. Finally, our findings are based on self-reported data, which might be subjected to common method variance (CMV) issues (Podsakoff et al., 2012). Although we have employed several methods to reduce CMV (e.g., aggregation of individual responses and respondent confidentiality), and this problem may not be seen being completely eliminated. To better avoid potential CMV problems, we suggest that future researchers could incorporate multi-wave or multi-source data.

## Data Availability

The raw data supporting the conclusions of this manuscript will be made available by the authors, without undue reservation, to any qualified researcher.

## Ethics Statement

This study was conducted in accordance with the ethical guidelines of the Institutional Review Board of Zhejiang University of Technology (ZJUT) in China, with written informed consent from all subjects. All the employees participated in the survey voluntarily. The protocol was approved by the Institutional Review Board of ZJUT and the Secretariat of Academic Committee of ZJUT, with the permit number 2019002.

## Author Contributions

TR and MR were responsible for data collection. All authors contributed equally to formulating the conceptual framework, analyzing the data, and writing the manuscript.

### Conflict of Interest Statement

The authors declare that the research was conducted in the absence of any commercial or financial relationships that could be construed as a potential conflict of interest.
